# Comparative characterization of bacterial communities in geese consuming of different proportions of ryegrass

**DOI:** 10.1371/journal.pone.0223445

**Published:** 2019-10-25

**Authors:** Baodi Guo, Dianhui Li, Beibei Zhou, Yong Jiang, Hao Bai, Yang Zhang, Qi Xu, Wenming Zhao, Guohong Chen

**Affiliations:** 1 Laboratory of Animal Genetics and Rearing and Molecular Design of Jiangsu Province, Yangzhou University, Yangzhou, PR, China; 2 Joint International Research Laboratory of Agriculture and Agri-Product Safety, the Ministry of Education of China, Institutes of Agricultural Science and Technology Development, Yangzhou University, Jiangsu Yangzhou, China; Washington State University - Spokane, UNITED STATES

## Abstract

Geese are extremely well-adapted to utilizing plant-derived roughage in their diet, so the grass must be added to commercial diets under intensive rearing systems. However, it is unclear whether the gut microbiota will change significantly when adding different proportions of ryegrass. In this study, 240 healthy male Yangzhou geese (28 days old) with similar body weights were randomly divided into four groups and fed different proportions grass (CK, whole commercial diets; EG1, ryegrass: commercial diets = 1.5:1; EG2, ryegrass: commercial diets = 2:1; EG3, ryegrass: commercial diets = 3:1) respectively. When the geese grew to 70 days old, their intestines were collected and high-throughput sequencing technology was performed to investigate the microbial diversity in the caecum of geese with different dietary supplements. There was no obvious change in the alpha diversity of gut microbiota of geese with ryegrass intake (*P* > 0.05) and the composition of dominant bacterium (including Bacteroidetes and Firmicutes) was also similar. However, the ratio between Firmicutes and Bacteroidetes was remarkably reduced with ryegrass intake (*P* < 0.05), and the relative abundance of 30 operational taxonomic units (OTUs) significantly differed. Additionally, the content of cellulose-degrading microbiota such as *Ruminiclostridium* and *Ruminococcaceae UCG-010* were significantly increased in geese fed with increasing amounts of grass. Finally, the functional profiles of the goose gut microbiota were explored using the PICRUSt tool. Carbohydrate metabolism and amino acid metabolism were dominant metabolic pathways. Lipid metabolism was significantly increased in EG3 compared that in the CK group (*P* < 0.05). Interestingly, *Turicibacter* and *Parasutterella* may have affected abdominal fat deposition as grass intake increased. Taken together, although the diversity of bacterial communities was similar in geese fed with different proportions of ryegrass, cellulose-degrading microbiota (*Ruminiclostridium* and *Ruminococcaceae UCG-010*) were abundant and the lipid metabolic pathway was enriched, which may reduce abdominal fat accumulation in high-ryegrass fed geese.

## Introduction

The gut microbiota colonizes the host intestine through a very complex process of interactions between the microbiota and their hosts [1]. Microbiota coevolve with the host and play a central role in multiple host functions such as metabolism, immunity, development, and behavior [2]. Most studies have evaluated the microbiota in humans and vertebrates, clearly demonstrating that the composition of gut microbiota is impacted by multiple environmental factors such as diet. This provides the opportunity to improve the physiological status of the host by regulating intestinal bacteria [3, 4]. In humans, diets enriched with fat and protein can increase the number of Firmicutes in gut microbiota compared to plant-based diets that are higher in fiber and increase the numbers of Bacteroidetes [4]. *Lachnospira* and *Ruminococcus* degrade pectin in humans and are vital for the colonic fermentation of dietary fibers [5]. Similarly, feeding of a high-fat diet increased the number of Firmicutes (Lactococcus), while the number of Bacteroidetes was decreased in mice fecal samples [6]. However, few studies have evaluated the intestinal microbiota in geese.

The goose (*Anas cygnoides*) is an important herbivore that supplies nutritious meat to humans [7]. Traditionally, geese are fed with grazing grass or cradling grass on a small-scale [8]. Using the original mode of feeding in intensive rearing of geese is impractical because of the high labor costs and land resources use. Therefore, feeding geese with commercial diets supplementing with a certain amount of grass has become the main method of production in the goose industry.

Goose has a strong ability to use coarse fiber from the grass but do not contain cellulolytic enzymes and hemi-cellulolytic enzymes, which are mainly secreted by microorganisms in the digestive tract. Therefore, it is important to study the diversity of gut microbes and microbiota composition. Xu et al. demonstrated that Bacteroidaceae and Lachnospiraceae are central cellulose-degrading microbiota [9]. By adding forage to the diet, the number of microorganisms that can degrade cellulose increased, such as *Selenomonadales* order, *Negativicutes* class, and *Oscillospir* and *Megamonas* genera [10]. However, the effects of different proportions of grass on the microbiota remain unclear.

Further, adding green grass to the diet influences the deposition of abdominal fat in geese [11]. Some microbiota can modulate obesity, such as the obesity-associated bacterial genus *Turicibacter* in rats [12] and *Mucispirillum schaedleri* and *Methanobrevibacter*, which are significantly correlated with fat deposition in chicken [13]. The microbiota associated with fat deposition in geese are not well-understood.

In this study, we performed 16S rRNA gene sequencing of geese consuming of different proportions of ryegrass, and the bacterial communities were characterized. Besides, the cellulose-degrading microbiota were identified abundantly in high-ryegrass fed geese, and the lipid metabolic pathway was enriched based on differentially represented OTUs. These data revealed the effect of the ryegrass addition on gut microbiota in geese.

## Materials and methods

### Ethics statement

All experimental protocols involving animals were approved by the Institutional Animal Care and Use Committee of Yangzhou University (approval number:151–2014). Procedures were performed in accordance with the Regulations for the Administration of Affairs Concerning Experimental Animals (Yangzhou University, China, 2012) and Standards for the Administration of Experimental Practices (Jiangsu, China, 2008).

### Sample collection

A total of 240 healthy male Yangzhou geese (28 days old) with similar body weights were randomly divided into four groups (CK, whole commercial diets; EG1, ryegrass: commercial diets = 1.5:1; EG2, ryegrass: commercial diets = 2:1; EG3, ryegrass: commercial diets = 3:1), and fed with different proportions of grass from 29 to 70 days of age (Table 1). The ingredient and nutrient levels of the commercial diets are shown in Table 2. After 12 h of starvation at 70 days old, all geese were weighed individually, and five geese per group (with body weight closest to the mean per group weight) were selected and sacrificed. Birds were slaughtered by manual exsanguination immediately by anesthetizing them with sodium pentobarbital. The contents of the caecum were aseptically removed, placed in a sterile tube, and immediately stored at -80°C until further analyses.

**Table 1:** Geese fed with different ratio of ryegrass addition to commercial diets.

Simple	CK	EG1	EG2	EG3
15~28 days old	Commercial diets	Commercial diets	Commercial diets	Commercial diets
29~70 days old	Commercial diets	Ryegrass: Commercial diets = 1.5:1	Ryegrass: Commercial diets = 2.0:1	Ryegrass: Commercial diets = 3.0:1

**Table 2:** Ingredient and nutrient levels of the commercial diets in geese (29–70 days).

Items	Content
Ingredients, %	
Corn	56
Soybean meal	21
Wheat bran	15
Premix	5
Bone meal	3
Nutrient levels	
Crude protein, %	17.2
Crude fat, %	3.7
Crude fiber, %	5.3
Ca, g/kg	10.7
Total P, g/kg	4.8
Lys, g/kg	7.6
Met, g/kg	4.4
Apparent ME, KJ/kg	10.46

Note: Premix provided per kilogram of diet: vitamin A, 2000 IU; vitamin D3, 45000U; vitamin E, 300IU; vitamin K3, 20 mg; vitamin B1, 10 mg; vitamin B2, 120 mg; vitamin B6, 20mg; nicotinic acid, 600 mg; pantothenic acid, 180 mg; folic acid, 10 mg; choline, 7 g; Fe, 1.2 g; Cu, 0.2 g; Mn, 1.9 g; Zn, 1.8 g; I, 10 mg; Se, 6 mg.

### DNA extraction and 16S rRNA gene sequencing

Microbiota genomic DNA was extracted from the samples using a Stool DNA Kit (OMEGA Bio-Tek, Norcross, GA, USA) according to the manufacturer’s instructions. The V4-V5 regions of the bacterial 16S rRNA gene were amplified by PCR (initial denaturation step at 98°C for 30 s, amplification at 98°C for 15 s, annealing at 50°C for 30 s, and extension at 72°C for 30 s, with an extra extension step at 72°C for 10 min) using the primers 515F: (5′-GTGCCAGCMGCCGCGG-3′) and 907R (5′-CCGTCAATTCMTTTRAGTTT-3′). PCR was performed in a 20-μL reaction volume containing 0.8 μL of each primer, 4 μL 5×FastPfu buffer, 2 μL 2.5mM dNTPs, 10 ng template DNA, and 0.4 μL FastPfu polymerase. Amplicons were extracted from 2% agarose gels and purified using an AxyPrep DNA Gel Extraction Kit (Axygen Biosciences, Union City, CA, USA) according to the manufacturer’s instructions and quantified using QuantiFluor^™^ -ST (Promega, USA). Next-generation sequencing was performed with an Illumina Miseq 2500 PE250 (San Diego, CA, USA) according to standard protocols provided by Biozeron, Inc. (Shanghai, China).

### Library construction and sequencing

Purified PCR products were quantified with a Qubit®3.0 (Life Invitrogen) and every 24 amplicons whose barcodes differed were mixed equally. The pooled DNA product was used to construct an Illumina Pair-End library following Illumina’s genomic DNA library preparation procedure. The amplicon library was paired-end sequenced (2 × 250) on an Illumina MiSeq platform according to standard protocols. The raw reads were deposited into the NCBI Sequence Read Archive database (Accession Number: PRJNA553112).

### Processing of sequencing data

Raw fastq files were demultiplexed, quality-filtered using QIIME (version 1.17) with the following criteria: (i) The 300-base pair (bp) reads were truncated at any site with an average quality score <20 over a 50-bp sliding window, discarding the truncated reads shorter than 10 bp. (ii) Exact barcode matching, 2 nucleotide mismatch in primer matching, and reads containing ambiguous characters were removed. (iii) Only sequences with overlaps longer than 10 bp were assembled according to their overlap sequence. Reads which could not be assembled were discarded.

OTUs were clustered with 97% similarity cutoff using UPARSE (version 7.1 http://drive5.com/uparse/) and chimeric sequences were identified and removed using UCHIME. The phylogenetic affiliation of each 16S rRNA gene sequence was analyzed by RDP Classifier (http://rdp.cme.msu.edu/) against the silva (SSU123) 16S rRNA database using confidence threshold of 70%.

### Bioinformatics and statistical analysis

Heatmaps were generated using R (http://www.r-project.org/) for the four groups. The functional profiles of the microbiota were predicted using PICRUSt. Species richness and alpha diversity were determined by Kruskal-Wallis analysis. Abdominal fat deposition percentage was analyzed by one-way analysis of variance. The Kruskal-Wallis and analysis of variance tests were performed using SPSS 17.0 software (SPSS, Inc., Chicago, IL, USA). *P* < 0.05 was considered statistically significant.

## Results

### Richness and diversity in caecal microbiota of geese fed with different proportions of ryegrass addition

A total of 1,053,761 qualified sequences were obtained from 20 samples. Data analysis revealed that each sample contained an average of 2,158 OTUs. We then compared microbial diversity, assessed diversity by the Simpson and Shannon indices, and estimated richness by determining the Chao index in the four groups. Although we found no significant differences in richness and diversity (*P* > 0.05), the Chao index increased with ryegrass intake in the diet (S1 Table). The coverage index was greater than 0.99 in each group.

### Dominance of Bacteroidetes and Firmicutes in geese fed with different propotion of ryegrass addition

Five major bacteria phyla were detected, Bacteroidetes, Firmicutes, Deferribacteres, Proteobacteria, and Cyanobacteria, among which Bacteroidetes and Firmicutes were dominant in each group (S1 Fig). Relevant differences were observed in the two predominant phyla: Bacteroidetes was less abundant in the CK group (60.27%) than in the EG1 (64.61%), EG2 (66.82%), and EG3 (65.71%) groups, whereas the relative abundance of Firmicutes was higher in the CK group (30.45%) than in the EG1 (27.83%), EG2 (26.19%), and EG3 (26.35%) groups (S2 Table). Statistical analysis by the Kruskal-Wallis test indicated that the Firmicutes to Bacteroidetes ratio was higher in the CK group and dramatically different from in the EG3 group (*P* < 0.05). Notably, the genera *Bacteroides*, *Prevotellaceae Ga6A1 group*, *Muribaculaceae_norank* (Bacteroidetes), *Faecalibacterium*, *Megamonas*, and *Ruminococcaceae_uncultured* (Firmicutes) were dominant in the four groups (S2 Fig).

### Identification of cellulose-degrading microbiota of geese fed with different proportion of ryegrass addition

**Fig 1:**
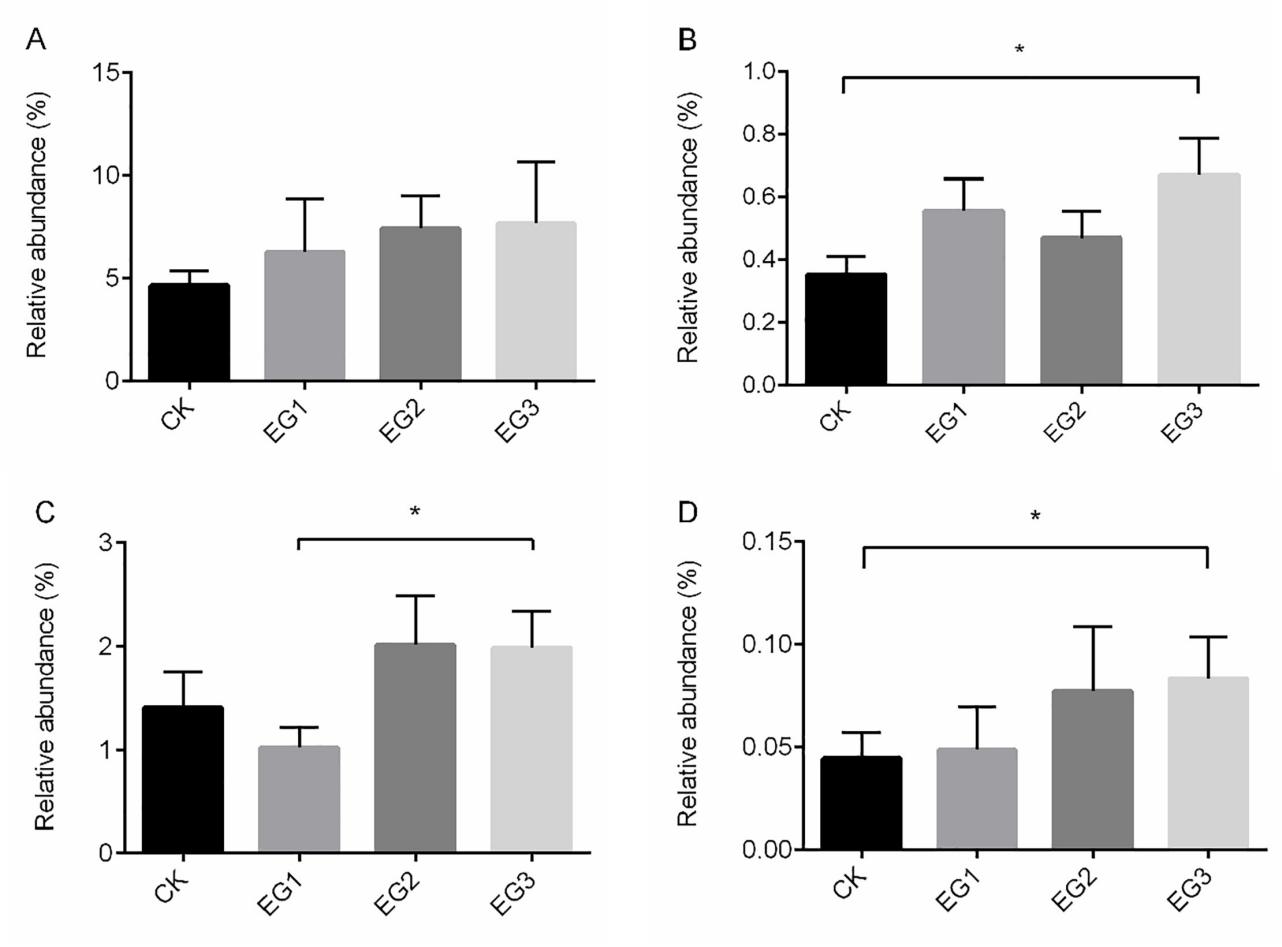
Different bacteria associated with cellulose-degrading in genera of geese. A, Prevotellaceae Ga6A1 group; B, Ruminiclostridium; C, Alistipes; D, Ruminococcaceae UCG-010. * represent significantly different at *P* < 0.05.

Ryegrass contains a large amount of crude fiber as the main dietary source of non-digestible carbohydrates. To identify significantly different bacteria associated with cellulose-degrading microbiota, we compared the caecum microbiota in the CK, EG1, EG2, and EG3 groups by the Kruskal-Wallis test. At the genus level, the relative abundances of *Prevotellaceae Ga6A1* group, *Ruminiclostridium*, *Alistipes*, and *Ruminococcaceae UCG-010* were influenced by diet. Although the *Prevotellaceae Ga6A1* group did not significantly differ between groups (*P* > 0.05), its relative abundance increased with increasing ryegrass intake (Fig 1A). *Alistipes* showed the lowest level in the EG1 group and highest level in the EG3 group (*P* < 0.05; Fig 1C). *Ruminiclostridium* and *Ruminococcaceae UCG-010* were also underrepresented in the CK group compared to in the EG3 group (*P* < 0.05; Fig 1B and 1D).

**Fig 2:**
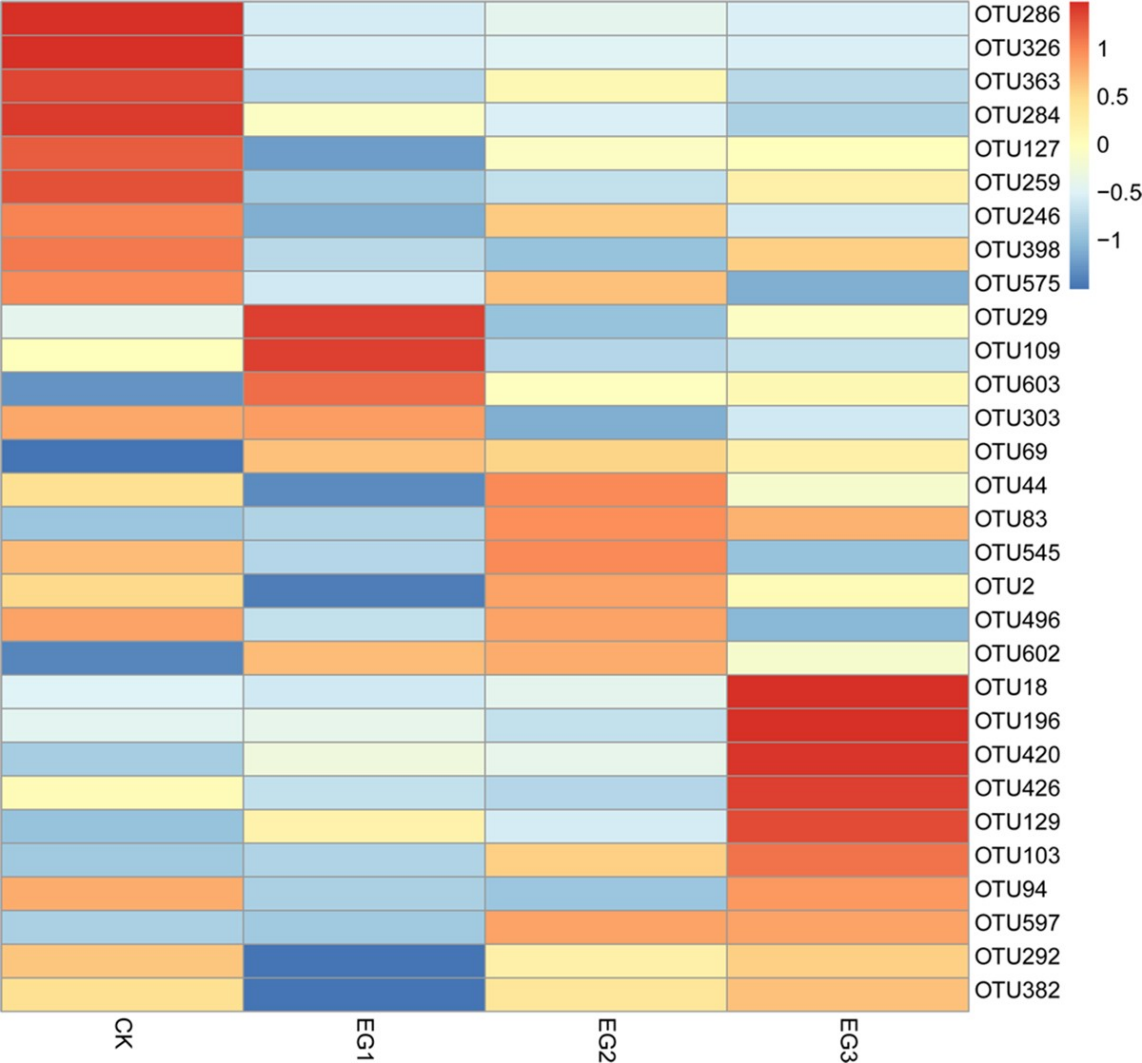
Heatmap of significant differences OUTs in geese fed with different propotion of ryegrass addition.

We also identified significant differences using the Kruskal-Wallis test in the CK, EG1, EG2, and EG3 groups. Notably, 30 OTUs were significantly different between groups (*P* < 0.05; S3 Table). The heatmap showed that OTU 286, OTU 326, OTU363, and OTU 284 were enriched in the CK group, while OTU18, OTU196, OTU420, and OTU426 were important in the EG3 group, which belonged to the families Ruminococcaceae, Prevotellaceae, and Bacteriodaceae (Fig 2).

### Relationship between bacterial communities and abdominal fat percentage in geese

**Fig 3:**
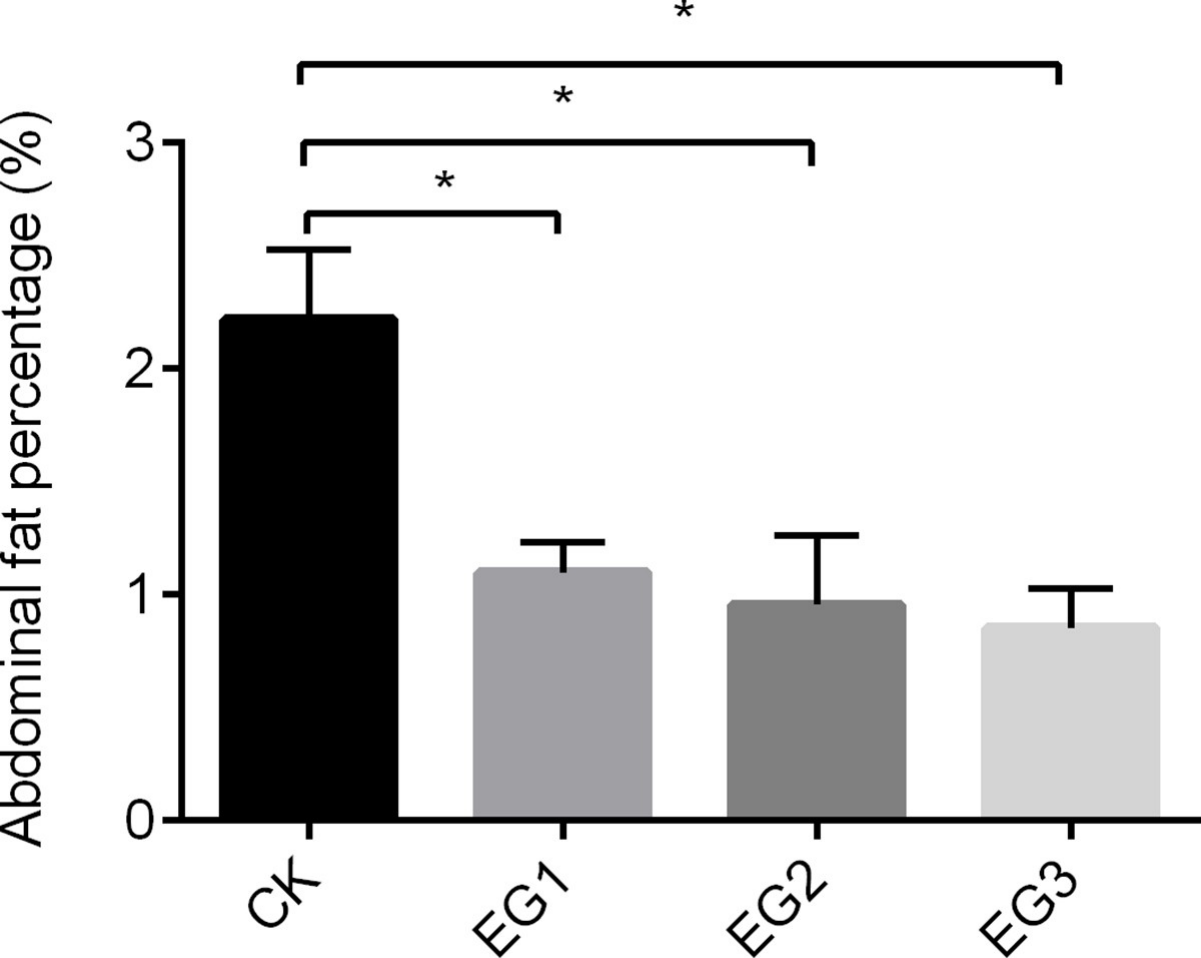
Abdominal fat percentage in geese fed with different proportions of ryegrass addition. * indicates significantly difference at *P* < 0.05.

We found that the abdominal fat percentage decreased gradually with ryegrass intake and was highest in the CK group, showing significant differences from the EC1, EG2, and EG3 groups (*P* < 0.05); however, there was no significant differences among the EC1, EC2, and EC3 groups (*P* < 0.05; Fig 3). We also found that *Barnesiella* and *Harryflintia* were negatively correlated with abdominal fat percentage, while some microbiota showed positive correlations such as *Turicibacter* and *Parasutterella* (Table 3).

**Table 3:** Relationship between bacterial communities and abdominal fat percentage in geese.

Bacteria	R	*P* value
*Barnesiella*	-0.58	0.02
*Harryflintia*	-0.52	0.04
*Turicibacter*	0.77	< 0.01
*Anaerobiospirillum*	0.64	0.01
*CHKCI001*	0.62	0.01
*Parasutterella*	0.59	0.02
*Ruminiclostridium*.*9*	0.58	0.02
*WCHB1*.*41_norank*	0.56	0.02
*Romboutsia*	0.53	0.03
*Lactobacillus*	0.52	0.04
*Lachnospiraceae_uncultured*	0.52	0.04
*Prevotella*.*9*	0.51	0.04
*Roseburia*	0.50	0.05
*Phascolarctobacterium*	0.50	0.05
*Family*.*XIII*.*UCG*.*001*	0.50	0.05
*Rhodospirillales_norank*	0.50	0.05
*Coriobacteriales*.*Incertae*.*Sedis_uncultured*	0.50	0.05
*Holdemania*	0.50	0.05
*Staphylococcus*	0.49	0.05

Note: Different superscripts differ significantly (*P* < 0.05).

### Function profiling of caecum microbiota in geese fed with different proportion of ryegrass addition

We used PICRUSt (Phylogenetic Investigation of Communities by Reconstruction of Unobserved States) as a tool for functional predictive exploration. The results showed that 40 of the 43 level II orthology groups (KOs in Kyoto Encyclopedia of Genes and Genomes) were detected in the Yangzhou goose caecum microbiome (S3 Fig). The dominant functions were carbohydrate metabolism, amino acid metabolism, replication and repair, membrane transport, and energy metabolism in the four groups. Lipid metabolism showed a high abundance suggesting a dramatic difference between the two groups (*P* < 0.05; Table 4).

**Table 4:** Lipid metabolism abundance in geese fed with different proportion of ryegrass addition.

Metabolism	CK	EG1	EG2	EG3	*P* value
Lipid Metabolism	362253 ± 73425^b^	415930 ± 91909^ab^	435712 ± 64043^ab^	484874 ± 61824^a^	0.048

Note: Different superscripts differ significantly (*P* < 0.05).

## Discussion

Geese, as herbivores, digest green forage to regulate their gut microbiota. Various factors may affect these bacteria, including genotypes, antibiotic factors, and environmental factors; particularly, the diet is a central factor influencing the gut microbiota [14, 15]. Fewer studies have evaluated the effect of adding different proportions of grass on the microorganisms in the goose gut. In this study, we characterized the gut microbiota in geese.

Exposure to the abundant bacteria associated with a high-fiber diet can enrich microbiome diversity [16]. High alpha diversity within the microbiota has been associated with good health, while low alpha diversity is related to poor health in humans [17]. In this study, we found that bacteria richness increased when ryegrass was added to the diet. Thus, ryegrass can be added during intensive goose rearing to improve goose health. The Shannon index was previously found to range from 4 to 5 among poultry [13] but varied from 7 to 9 in rabbit [18], goat [19], and swine [20]. In this study, the average Shannon index was approximately 5, which was similar to the value in chicken.

Most studies have focused on the microbiota in humans and vertebrates [21]. Ley et al. showed that more than 94.2% of sequences in all Burkina Faso and European samples belonged to two major populated microbiota phyla: Actinobacteria and Bacteroidetes [21]. Velasco-Galilea et al. found that the predominant bacteria were Firmicutes (76.4%) and Tenericutes (7.8%) in the caecum of rabbit [22]. Firmicutes and Proteobacteria account for most of the bacteria in black bears [23]. However, in this study, Yangzhou geese were dominantly occupied by Bacteroidetes and Firmicutes. Bacteroidetes is associated with the degradation of cellulose [24]. We found that the relative abundance of Bacteroidetes increased with increasing ryegrass in the diets. Firmicutes, an important phylum, is involved in protein and fat metabolism [25]. A diet consisting of approximately 30% calories from protein and 70% from fat results in enrichment of Firmicutes in human [26]. Our results showed that Firmicutes, which was the second most abundant bacterium, was decreased (from 30.45% to 26.19%) with increasing ryegrass intake, likely because commercial diets contained more calories than ryegrass. Furthermore, the relative proportion of Bacteroidetes increases while Firmicutes decreases in humans with increased crude fiber intake in the diet [27]. Our results showed that the Firmicutes/Bacteroidetes ratio was decreased with increasing ryegrass intake.

Grass is a source of dietary fiber that must be fermented by microbiota in the caecum to produce energy. Prevotellaceae was reported as the most abundant microbiota in bovine and goats, both of which have a powerful ability to digest crude fibers [28]. Here, we found that the abundance of *Prevotellaceae Ga6A1* group (family Prevotellaceae) was increased with increasing ryegrass in the diet. *Ruminiclostridium* can depolymerize cellulose and related plant cell wall polysaccharides [29]. The *Ruminococcaceae* family is important for degrading pectin and cellulose in the colonic fermentation of dietary fibers [30]. When humans switch from an animal to plant-based diet, the relative abundance of *Alistipes* was found to be increased [31]. In this study, *Ruminiclostridium*, *Ruminococcaceae UCG-010* (family *Ruminococcaceae*), and *Alistipes* were increased, indicating their association with digestive fibers. Interestingly, OTU18, OTU196, OTU420, and OTU426 were major in the EG3 group and belong to the families Ruminococcaceae, Prevotellaceae, and Bacteriodaceae. Ruminococcaceae and Prevotellaceae are related to fiber digestion in the rumen [28, 32], which Bacteroidaceae is a central cellulose-degrading microbiota in geese [9]. Therefore, adding ryegrass to the diet can increase the relative abundance of cellulose-degrading bacteria.

Accumulating studies have demonstrated that obesity is associated with an imbalance in the normal bacteria composition [33]. Bacteria influence whole-body metabolism by impacting the energy balance associated with obesity [34]. *Barnesiella* is related to carbohydrate utilization [35] and *Harryflintia* belongs to the family Ruminococcaceae, which is associated with fiber digestion [30]. In this study, we found that *Barnesiella* and *Harryflintia* were negatively correlated with the abdominal fat percentage. The degradation process of ryegrass may require bacteria involved in cellulose and carbohydrate metabolism. Furthermore, high-grain feeding increases the abundance of *Turicibacter* in goats [19]. *Parasutterella* was elevated by sugar consumption in rat [36]. Our results demonstrate that *Turicibacter* and *Parasutterella* were positively correlated with the abdominal fat percentage. Because these bacteria break down higher-energy foods, they can increase abdominal fat deposition.

Furthermore, the caecum microbiota plays an important role in host metabolic pathways. Our results demonstrated that carbohydrate metabolism and amino acid metabolism were dominant functions in the four groups. Because the main ingredients in feed are carbohydrate and protein, the main metabolic pathways may be carbohydrate metabolism and amino acid metabolism in geese. Previous studies reported that carbohydrate metabolism and amino acid metabolism were the most abundant functional categories in geese [37]. We also found that enrichment of the lipid metabolic pathway decreased the accumulation of abdominal fat when grass was added to the diet. PICRUSt provided insight into the function of intestinal bacterial communities in geese. Additional histological methods (such as transcriptome and metabolomics) should be used to improve our understanding of bacterial function in the goose caecum.

## Conclusions

Our study revealed the overall composition of the microbiota in the caecum of geese that consumed different diets based on 16S rRNA gene sequencing. The diversity of bacterial communities was similar when geese were fed with different proportions of ryegrass, However, cellulose-degrading microbiota such as *Ruminiclostridium* and *Ruminococcaceae UCG-010* were clearly observed in high-ryegrass fed geese. The lipid metabolic pathway was enriched based on differentially represented OTUs, which may reduce abdominal fat accumulation. These data revealed the effect of ryegrass addition on gut microbiota in geese, which providing a theoretical basis for adding an appropriate proportion of grass to the diet during intensive geese breeding.

## Supporting information

S1 Table Diversity estimation of the 16S rRNA gene libraries of the goose caecum from sequencing analysis. Note: The richness estimators (Chao) and diversity indices (Shannon and Simpson) were calculated. Coverage refers to the coverage of the sample libraries. A higher the value indicated a higher the probability that the sequence in the sample is detected and lower probability that the sequence was not detected. Samples in the CK: geese were fed with commercial diets diet. EG1-EG3: geese were fed with commercial diet plus different proportions of fresh ryegrass from 29 to 70 days old,; proportions of ryegrass to commercial diets were 1.5:1, 2:1 and 3:1, respectively.
(DOCX)

S2 Table Significantly different bacterial communities at phylum level in geese fed with different proportion of ryegrass addition. (DOCX)

S3 Table Significant differences OTUs in geese fed with different proportion of ryegrass addition. (DOCX)

S1 Fig Taxonomic profiles of the microbial communities at the phylum level of geese fed with different proportion of ryegrass addition. (TIF)

S2 Fig Pie charts of median percent values of bacterial genera present in caecal samples of geese fed with different proportion of ryegrass addition (>3%). (A), caecal samples in CK group; (B), caecal samples in EG1 group; (C), caecal samples in EG2 group; (D), caecal samples in EG3 group.
(TIF)

S3 Fig Functional predictions of all samples using PICRUSt. Using PICRUSt as a predictive exploratory tool, comparing overall 40 level 2 KEGG Orthology groups (KOs) represented in data set among geese samples from four groups.
(TIF)
