# Supplementary material for: Comparative characterization of bacterial communities in geese consuming of different proportions of ryegrass
Source: PLoS One. 2019 Oct 25;14(10):e0223445. doi: 10.1371/journal.pone.0223445 (PMC6814310; doi:10.1371/journal.pone.0223445)
Supplement: S1 Table — Note: The richness estimators (Chao) and diversity indices (Shannon and Simpson) were calculated. Coverage refers to the coverage of the sample libraries. A higher the value indicated a higher the probability that the sequence in the sample is detected and lower probability that the sequence was not detected. Samples in the CK: geese were fed with commercial diets diet. EG1-EG3: geese were fed with commercial diet plus different proportions of fresh ryegrass from 29 to 70 days old,; proportions of ryegrass to commercial diets were 1.5:1, 2:1 and 3:1, respectively. (DOCX) [file pone.0223445.s001.docx]

| **Index** | **CK** | **EG 1** | **EG 2** | **EG 3** | ***P* value** |
| --- | --- | --- | --- | --- | --- |
| Chao | 1486.824 ± 88.759 | 1489.632 ± 280.618 | 1589.968 ± 138.619 | 1542.814 ± 90.756 | 0.653 |
| Shannon | 5.026 ± 0.105 | 4.926 ± 0.456 | 5.075 ± 0.285 | 5.045 ± 0.182 | 0.951 |
| Simpson | 0.021 ± 0.006 | 0.025 ± 0.013 | 0.02 ± 0.008 | 0.019 ± 0.007 | 0.772 |
| Coverage | 0.992 ± 0.003 | 0.993 ± 0.001 | 0.993 ± 0.001 | 0.993 ± 0.001 | 0.978 |
