# Supplementary material for: Comparative characterization of bacterial communities in geese consuming of different proportions of ryegrass
Source: PLoS One. 2019 Oct 25;14(10):e0223445. doi: 10.1371/journal.pone.0223445 (PMC6814310; doi:10.1371/journal.pone.0223445)
Supplement: S2 Table — (DOCX) [file pone.0223445.s002.docx]

| **The phyla of bacteria** | **CK (%)** | **EG1 (%)** | **EG2 (%)** | **EG3 (%)** | ***P* value** |
| --- | --- | --- | --- | --- | --- |
| Firmicutes / Bacteroidetes | 0.508±0.074^a^ | 0.475±0.257^ab^ | 0.397±0.101^ab^ | 0.404±0.102^b^ | 0.047 |
| Actinobacteria | 0.516±0.254 | 0.348±0.146 | 0.59±0.493 | 0.505±0.279 | 0.785 |
| Bacteroidetes | 60.272±3.974 | 64.614±14.034 | 66.816±4.962 | 65.71±4.333 | 0.287 |
| Cyanobacteria | 1.698±0.677 | 1.13±1.068 | 1.545±1.124 | 1.192±0.535 | 0.351 |
| Deferribacteres | 2.892±3.359 | 2.523±2.973 | 1.567±1.893 | 2.277±4.201 | 0.754 |
| Elusimicrobia | 0.497±0.341 | 0.098±0.085 | 0.277±0.213 | 0.432±0.415 | 0.082 |
| Epsilonbacteraeota | 0.003±0.003 | 0.011±0.015 | 0.004±0.004 | 0.001±0.002 | 0.542 |
| Euryarchaeota | 0.108±0.156 | 0.292±0.41 | 0.132±0.168 | 0.344±0.496 | 0.813 |
| Firmicutes | 30.446±3.312 | 27.833±9.681 | 26.189±4.78 | 26.352±5.449 | 0.448 |
| Fusobacteria | 0.014±0.029 | 0.407±0.645 | 0.099±0.189 | 0.017±0.028 | 0.487 |
| Kiritimatiellaeota | 0.227±0.341 | 0.016±0.019 | 0.119±0.266 | 0.021±0.025 | 0.724 |
| Lentisphaerae | 0.067±0.067 | 0.033±0.023 | 0.031±0.022 | 0.077±0.105 | 0.817 |
| Proteobacteria | 2.162±1.135 | 1.741±1.117 | 1.458±0.851 | 1.463±1.016 | 0.809 |
| Spirochaetes | 0.165±0.036 | 0.208±0.286 | 0.138±0.146 | 0.243±0.139 | 0.487 |
| Synergistetes | 0.059±0.06 | 0.152±0.211 | 0.138±0.119 | 0.187±0.131 | 0.534 |
| Tenericutes | 0.824±0.449 | 0.572±0.497 | 0.882±0.596 | 1.161±0.824 | 0.524 |
| Unclassified | 0.003±0.004 | 0.002±0.003 | 0.001±0.002 | 0.003±0.006 | 0.854 |
| Verrucomicrobia | 0.049±0.056 | 0.018±0.011 | 0.014±0.019 | 0.016±0.01 | 0.487 |
