# Supplementary material for: Comparative characterization of bacterial communities in geese consuming of different proportions of ryegrass
Source: PLoS One. 2019 Oct 25;14(10):e0223445. doi: 10.1371/journal.pone.0223445 (PMC6814310; doi:10.1371/journal.pone.0223445)
Supplement: S3 Table — (DOCX) [file pone.0223445.s003.docx]

| **OTU** | **The families** of bacteria | **CK (%)** | **EG1 (%)** | **EG2 (%)** | **EG3 (%)** | ***P*** value |
| --- | --- | --- | --- | --- | --- | --- |
| OTU284 | Acidaminococcaceae | 21.4 ± 4.16 | 12.6 ± 8.26 | 9.8 ± 5.63 | 8 ± 3.54 | 0.033 |
| OTU2 | Bacteroidaceae | 1213 ± 1696.2 | 200 ± 126.67 | 1386 ± 1166.5 | 982.8 ± 704.59 | 0.003 |
| OTU44 | Bacteroidaceae | 200.2 ± 293.84 | 25.6 ± 12.6 | 251.4 ± 279.74 | 141 ± 96.89 | 0.025 |
| OTU127 | Bacteroidaceae | 79.8 ± 61.45 | 10.4 ± 9.1 | 43.4 ± 31.7 | 44.8 ± 16.08 | 0.037 |
| OTU246 | Bacteroidaceae | 30.8 ± 32.28 | 3 ± 2 | 25.2 ± 16.63 | 9.4 ± 7.4 | 0.01 |
| OTU426 | Bacteroidaceae | 7.6 ± 7.02 | 4 ± 4.24 | 3.6 ± 2.7 | 13.8 ± 6.61 | 0.039 |
| OTU575 | Bacteroidaceae | 7.6 ± 6.27 | 1.8 ± 1.3 | 6.4 ± 6.19 | 0 ± 0 | 0.027 |
| OTU602 | Bacteroidaceae | 0.2 ± 0.45 | 4.6 ± 5.13 | 4.8 ± 8.53 | 2.8 ± 2.49 | 0.034 |
| OTU292 | Barnesiellaceae | 18.6 ± 12.82 | 1.2 ± 1.64 | 15.2 ± 8.53 | 18.2 ± 12.05 | 0.042 |
| OTU326 | Erysipelotrichaceae | 31.6 ± 21.61 | 5 ± 3.46 | 5.6 ± 4.56 | 4.8 ± 1.92 | 0.012 |
| OTU196 | Prevotellaceae | 12.2 ± 13.05 | 13.4 ± 14.84 | 6.4 ± 6.5 | 60.2 ± 47.67 | 0.03 |
| OTU420 | Ribaculaceae | 2.2 ± 2.39 | 5.4 ± 2.3 | 4.8 ± 5.22 | 14.6 ± 7.6 | 0.022 |
| OTU109 | Rikenellaceae | 63.4 ± 141.77 | 170.2 ± 185.48 | 4.6 ± 10.29 | 11.2 ± 25.04 | 0.012 |
| OTU382 | Rikenellaceae | 9.6 ± 4.93 | 1.8 ± 2.05 | 9.2 ± 5.89 | 10.4 ± 9.71 | 0.047 |
| OTU398 | Rikenellaceae | 13.4 ± 11.01 | 4 ± 5.05 | 3 ± 2.74 | 10.8 ± 4.38 | 0.043 |
| OTU18 | Ruminococcaceae | 130.4 ± 171.43 | 97 ± 91.74 | 159.6 ± 165.45 | 788.2 ± 325.22 | 0.013 |
| OTU129 | Ruminococcaceae | 25 ± 8.8 | 50.6 ± 35.1 | 34.2 ± 15.32 | 76 ± 20.11 | 0.021 |
| OTU29 | Ruminococcaceae | 159.2 ± 60.16 | 312.4 ± 112.34 | 113.6 ± 59.56 | 189.8 ± 50.23 | 0.03 |
| OTU69 | Ruminococcaceae | 39 ± 6.82 | 112 ± 53.93 | 108.2 ± 71.9 | 96.4 ± 37.82 | 0.05 |
| OTU83 | Ruminococcaceae | 26 ± 16.48 | 32.8 ± 27.88 | 121.2 ± 119.13 | 110.4 ± 64.12 | 0.049 |
| OTU103 | Ruminococcaceae | 36.6 ± 4.98 | 39 ± 27.52 | 68.8 ± 25.19 | 80.4 ± 30.98 | 0.043 |
| OTU286 | Ruminococcaceae | 70.2 ± 130.94 | 0.2 ± 0.45 | 5.2 ± 10.52 | 1.4 ± 1.34 | 0.016 |
| OTU303 | Ruminococcaceae | 17.2 ± 15.09 | 17.6 ± 6.73 | 4.6 ± 5.5 | 8 ± 8.25 | 0.05 |
| OTU496 | Ruminococcaceae | 7.4 ± 7.13 | 2.2 ± 2.28 | 7.4 ± 3.91 | 1 ± 1.73 | 0.023 |
| OTU597 | Ruminococcaceae | 0.4 ± 0.55 | 0.2 ± 0.45 | 6.6 ± 3.58 | 6.6 ± 3.91 | 0.003 |
| OTU603 | Ruminococcaceae | 1 ± 0.71 | 5.2 ± 3.42 | 3.2 ± 1.64 | 3.4 ± 1.95 | 0.023 |
| OTU545 | Uncultured | 5.8 ± 3.96 | 2 ± 1.22 | 6.6 ± 5.03 | 1.6 ± 1.14 | 0.047 |
| OTU259 | Uncultured | 30.8 ± 23.06 | 4.6 ± 5.46 | 7.2 ± 6.98 | 17.8 ± 11.65 | 0.03 |
| OTU363 | Uncultured | 19.4 ± 15.36 | 2.2 ± 2.28 | 9.2 ± 7.76 | 2.4 ± 5.37 | 0.011 |
| OTU94 | Veillonellaceae | 110.6 ± 130.43 | 8.4 ± 9.29 | 2 ± 2.35 | 118.4 ± 249.29 | 0.035 |
