# Supplementary figures and images for: Comparative characterization of bacterial communities in geese consuming of different proportions of ryegrass
Source: PLoS One. 2019 Oct 25;14(10):e0223445. doi: 10.1371/journal.pone.0223445 (PMC6814310; doi:10.1371/journal.pone.0223445)

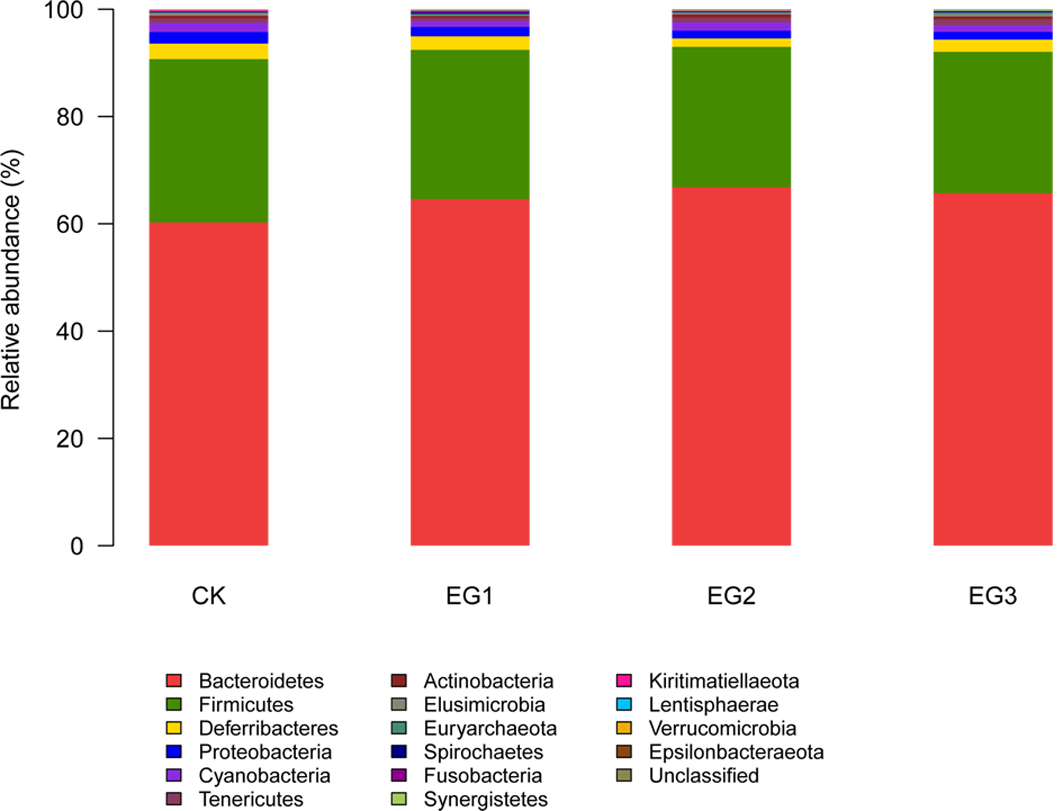

Supplement: S1 Fig — (TIF) [file pone.0223445.s004.tif]

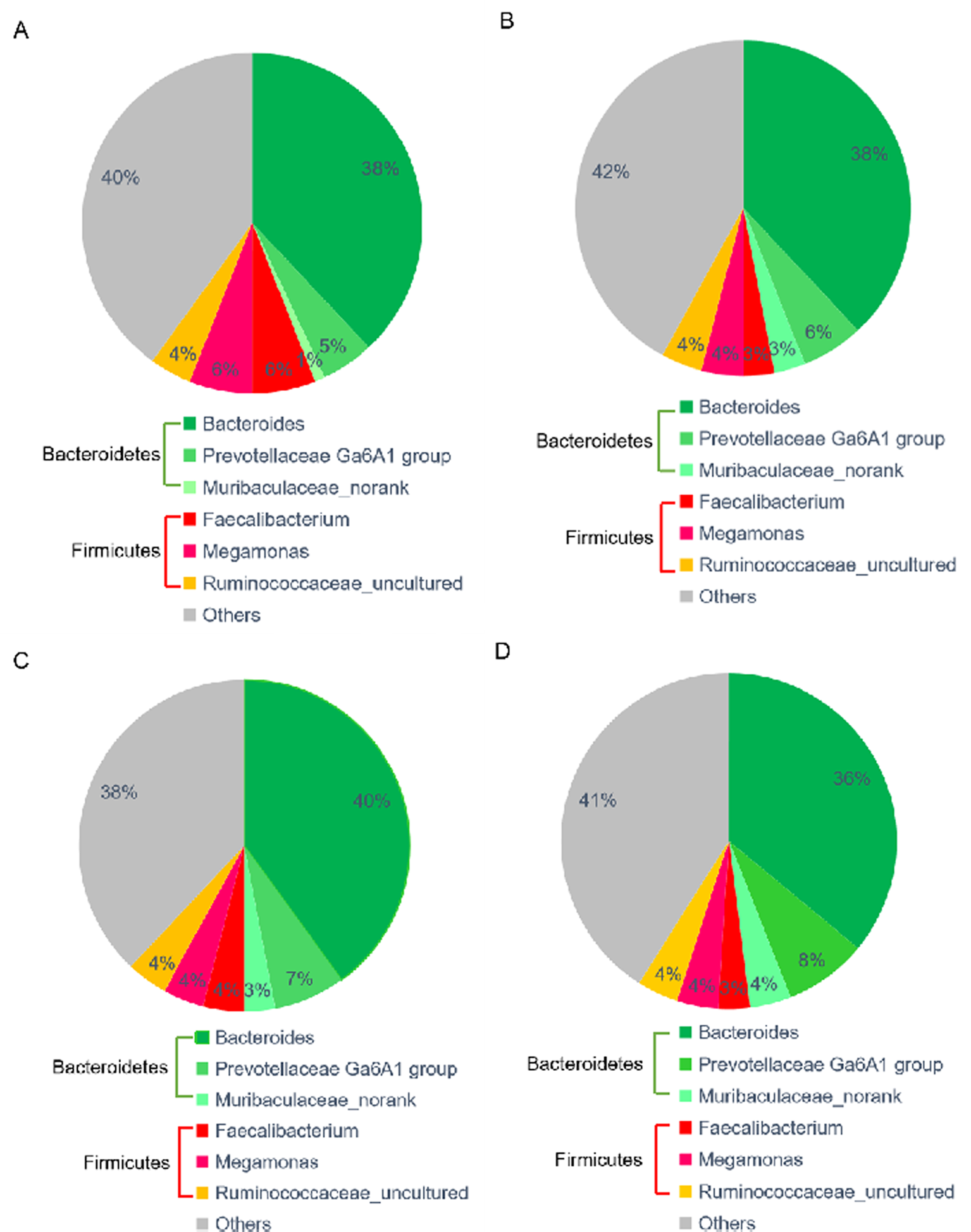

Supplement: S2 Fig — (A), caecal samples in CK group; (B), caecal samples in EG1 group; (C), caecal samples in EG2 group; (D), caecal samples in EG3 group. (TIF) [file pone.0223445.s005.tif]

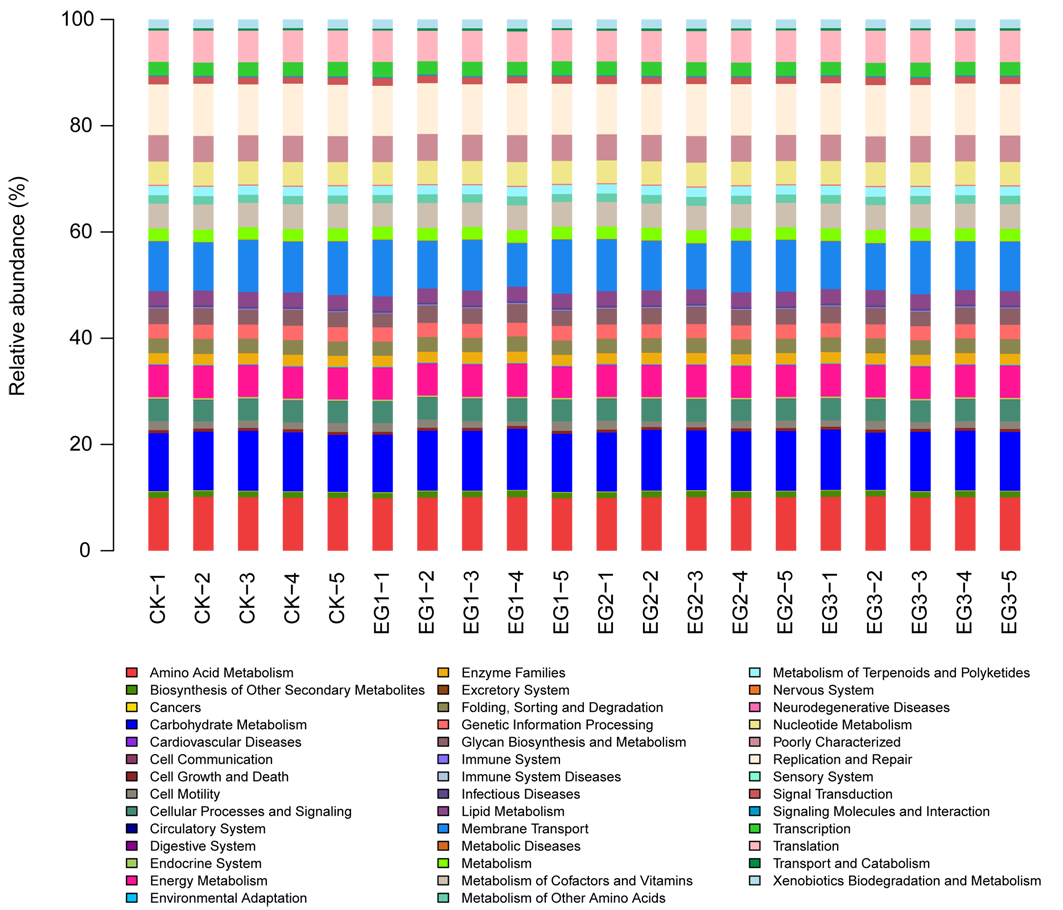

Supplement: S3 Fig — Using PICRUSt as a predictive exploratory tool, comparing overall 40 level 2 KEGG Orthology groups (KOs) represented in data set among geese samples from four groups. (TIF) [file pone.0223445.s006.tif]
